# Falcarindiol promotes beige adipocyte-related gene expression and mitochondrial respiration in human preadipocyte-derived adipocytes

**DOI:** 10.1007/s10616-025-00790-y

**Published:** 2025-06-16

**Authors:** Shingo Takahashi, Haruka Okaze, Seiji Kawamoto

**Affiliations:** 1 Diet & Well-being Research Institute, KAGOME CO., LTD., 17 Nishitomiya, Nasushiobara, Tochigi 329-2762 Japan; 2 Program of Biotechnology, Graduate School of Integrated Sciences for Life, Hiroshima University, 1-3-1 Kagamiyama, Higashi-Hiroshima, 739-8530 Japan

**Keywords:** Falcarindiol, Apiaceae, Adipocyte browning, PPARγ, UCP1, Obesity

## Abstract

Falcarindiol, a typical polyacetylene compound found in Apiaceae vegetables, activates peroxisome proliferator-activated receptor γ (PPARγ). However, whether it induces the browning of adipocytes through PPARγ activation is unclear. In this study, we aimed to clarify the effects of falcarindiol on adipocyte browning and mitochondrial respiration in human preadipocyte-derived adipocytes. Human primary cultured cells were differentiated for 8 days in the presence of falcarindiol. The expression of PPARγ target and beige adipocyte-related genes was measured using quantitative real-time PCR, and the accumulation of lipid droplets and uncoupling protein 1 (UCP1) protein expression were evaluated using immunohistochemistry. The oxygen consumption rate was measured using a Seahorse flux analyzer. Falcarindiol increased the expression of PPARγ target genes, including *PPARγ*, *FABP4*, *SLC2A4*, and *ADIPOQ*. It also increased the expression of beige adipocyte-related genes, such as *PPARGC1A*, *PPARA*, *CITED1*, and *TBX1*, and increased the expression of UCP1 protein. Falcarindiol also significantly increased basal respiration, ATP-linked respiration, maximal respiration, spare capacity, and proton-leak respiration, and significantly decreased the coupling efficiency in a concentration-dependent manner. These results indicate that falcarindiol promotes a beige adipocyte-like phenotype and oxygen consumption of adipocytes in vitro, suggesting that dietary intake of falcarindiol and falcarindiol-containing Apiaceae vegetables may be effective in obesity prevention.

## Introduction

Obesity is a serious global public health concern. The number of persons with obesity is expected to reach two billion, which is approximately 24% of the world population, and the impact of obesity and overweight on the global economy is expected to reach $4.32 trillion by 2035 (World Obesity Federation 2023). Obesity is also a risk factor for various chronic diseases, such as diabetes and cardiovascular diseases (Powell-Wiley et al. 2021; Klein et al. 2022). It is caused by multiple factors, including genetic and epigenetic factors, but the main factor is an imbalance between energy intake and metabolism (Romieu et al. 2017). Therefore, increasing energy expenditure is an effective strategy to prevent obesity. Adipose tissue regulates systemic energy metabolism. Mammalian adipose tissue is classified into two types: white adipose tissue (WAT) and brown adipose tissue. WAT stores energy as triglycerides, while brown adipose tissue dissipates energy through thermogenesis. Thermogenic adipocytes include classical brown and beige adipocytes, which have a different origin from brown adipocytes. Beige adipocytes have the same origin as white adipocytes and appear in WAT deposits. However, similar to brown adipocytes, they are characterized by small multilocular lipid droplets, high mitochondrial content, and expression of uncoupling protein 1 (UCP1) (Kajimura et al. 2015; Ikeda et al. 2018). UCP1 localizes in the mitochondrial inner membrane and causes proton leakage, releasing energy in the form of heat without ATP synthesis, resulting in energy dissipation (Chouchani et al. 2019). Therefore, the biosynthesis of beige adipocytes in WAT, referred to as browning, is considered to be a promising approach for the prevention of obesity. Browning is not only caused by cold exposure and exercise but also by long-term treatment with peroxisome proliferator-activated receptor γ (PPARγ) agonists (Ohno et al. 2012). Therefore, the promotion of browning through the consumption of ingredients with PPARγ agonist activity in daily food is an effective way to prevent obesity.

The Apiaceae family includes a wide variety of vegetables and medicinal plants (Wang et al. 2022). Apiaceae vegetables are widely consumed worldwide and contain a rich variety of secondary metabolites; therefore, the relationship between their consumption and health is of notable interest. Carrots are a typical Apiaceae vegetable, and their raw consumption is associated with a reduced risk of developing colorectal, lung, and pancreatic cancers, as well as leukemia (Deding et al. 2020, 2023). β-Carotene is well known as the functional ingredient in carrots, but recently, the falcarinol-type polyacetylenes, which are characteristic of Apiaceae plants, have attracted attention (Dawid et al. 2015). Falcarinol-type polyacetylenes are aliphatic C_17_-polyacetylenes, and typical examples include falcarinol, falcarindiol, and falcarindiol-3-acetate, which are found in vegetables, such as celeriac, parsley, parsnip, as well as carrot (Christensen and Brandt 2006; Christensen 2011). Falcarinol-type polyacetylenes are thought to prevent bacterial infection in plant bodies due to their antifungal activity (Christensen 2011), but their health benefits for humans have also been noted. In particular, falcarinol and falcarindiol have anti-inflammatory (Metzger et al. 2008; Venkatesan et al. 2018) and anti-cancer effects (Kobaek-Larsen et al. 2017, 2019; Alfurayhi et al. 2023); therefore, they may play a role in the health benefits associated with Apiaceae vegetables. Falcarindiol activates PPARγ and induces adipocyte differentiation (Atanasov et al. 2013; El-Houri et al. 2015). Additionally, a single dose of Japanese parsley extract containing falcarindiol significantly decreased blood glucose levels in rats, confirmed by an oral glucose tolerance test (Yoshida et al. 2013). Therefore, falcarindiol has anti-obesity effects; however, its effects on fat browning have not been clarified. To address this gap and better understand the mechanism underlying falcarindiol’s effect on obesity, we evaluated its ability to induce beige adipocyte phenotypes and its influence on mitochondrial respiration using human adipocytes.

## Materials and methods

### Chemicals and reagents

**Fig. 1:**
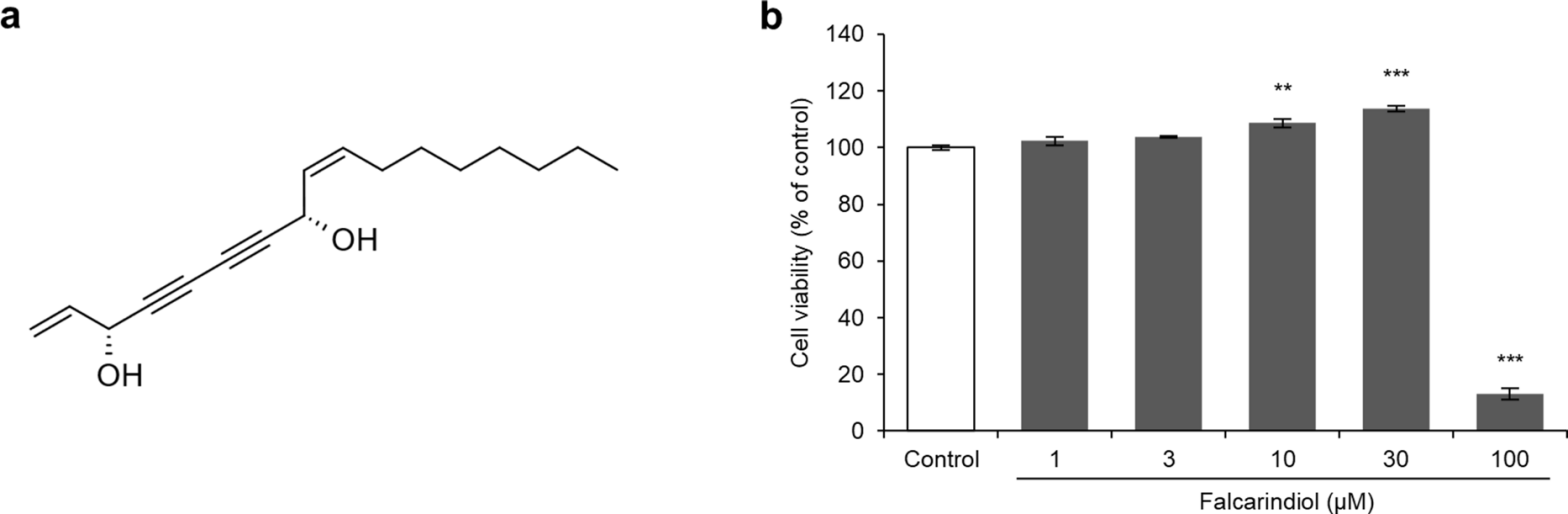
Effects of falcarindiol on human preadipocyte-derived adipocyte viability **a** Chemical structure of falcarindiol. **b** Viability of human preadipocyte-derived adipocytes. Human preadipocytes were differentiated into mature adipocytes for eight days in differentiation medium with or without falcarindiol. Cell viability was assessed using the WST-8 assay on day 8. Data are presented as mean ± SEM (n = 3). **p < 0.01 and ***p < 0.001 compared with the control (0.1% DMSO)

The (3*R*, 8*S*)-falcarindiol (Fig. 1a) with a purity ≥ 98% was purchased from ChemFaces (Wuhan, China). Trypsin–EDTA (0.05%, phenol red), Dulbecco's phosphate-buffered saline (DPBS; no calcium, no magnesium), Hoechst 33,342 (trihydrochloride trihydrate, 10 mg/mL), BODIPY 493/503, and goat anti-rabbit IgG (H + L) cross-adsorbed secondary antibody (Alexa Fluor 594, Cat No. A-11012) were purchased from Thermo Fisher Scientific (Waltham, MA, USA). Dimethyl sulfoxide (DMSO), cell count reagent SF, and 4% paraformaldehyde phosphate-buffered solution (PBS) were purchased from Nacalai Tesque (Kyoto, Japan). FastLane Cell cDNA Kit was purchased from QIAGEN (Hilden, Germany). Bovine serum albumin (BSA) was purchased from Sigma-Aldrich (St. Louis, MO, USA). Triton X-100 was purchased from MP Biomedicals (Santa Ana, CA, USA). Anti-UCP1 antibody (Cat No. ab10983) was purchased from Abcam (Cambridge, MA, USA). Seahorse XF Cell Mito Stress Test Kit, Seahorse XF base medium (phenol free), Seahorse XF 1.0 M glucose solution, Seahorse XF 100 mM pyruvate solution, Seahorse XF 200 mM glutamine solution, and Seahorse XFe96/XF Pro FluxPak were purchased from Agilent Technologies (Palo Alto, CA, USA).

### Cell culture and differentiation

Human subcutaneous preadipocyte cells (PT-5020, Lot No. 0000645827, 43 years old, female, Caucasian, body mass index 23 kg/m^2^) were purchased from Lonza (Walkersville, USA) and differentiated according to the manufacturer’s instructions using the PGM-2 Preadipocyte Growth Medium-2 BulletKit (PT-8002, Lonza). The cells were cultured in growth medium comprising Preadipocyte Basal Medium-2 (PBM-2; Lonza) supplemented with 10% fetal bovine serum, 2 mM L-glutamine, 50 μg/mL gentamycin, and 37 ng/mL amphotericin B. The culture medium was replaced every 1–2 days, and cells that had reached 80% confluence were used for differentiation. Preadipocytes were seeded at a density of 10,000 cells/well in a 96-well plate with growth medium, and after 1 day of incubation, the cells were induced for differentiation with or without falcarindiol using differentiation medium containing insulin, dexamethasone, indomethacin, and isobutyl-methylxanthine in growth medium. The cells were treated with falcarindiol or DMSO vehicle (0.1%) for a total of 8 days. Briefly, following the manufacturer's instructions, after 1 day of culture, 2 × differentiation medium adjusted to twice the target concentration of falcarindiol was added to the wells in equal amounts. The cells were then cultured for 8 days without changing the medium. Falcarindiol was dissolved in DMSO and added to the medium at a final concentration of 0.1% (v/v). All cell culture experiments were performed at 37 °C in a 5% CO_2_ humidified incubator (SCA-165DRS, Astec, Fukuoka, Japan).

### Cell viability and cytotoxicity assay

Cell viability was assessed using the WST-8 assay. After 8 days of differentiation with or without falcarindiol (1, 3, 10, 30, or 100 μM), the medium was replaced with differentiation medium containing a 1/10 dilution of cell count reagent SF. The plate was then incubated in a CO_2_ humidified incubator and the absorbance at 450 nm (reference wavelength: 630 nm) was measured using a microplate reader (VarioSkan Flash, Thermo Fisher Scientific) at 30 and 90 min. The difference in absorbance between 30 and 90 min was calculated, and cell viability was expressed as a percentage relative to that of the control (0.1% DMSO).

### Quantitative real-time PCR (qPCR)

The preadipocyte cells were differentiated with or without falcarindiol (1, 3, 10, or 30 μM), as described above. On day 8, total RNA was extracted from the cells using the FastLane Cell Probe Kit (QIAGEN) according to the manufacturer's protocol. One-step real-time RT-PCR was performed using TaqMan probes purchased from Thermo Fisher Scientific: *ACTB* (Hs01060665_g1), *ADIPOQ* (Adiponectin, Hs00977214_m1), *CIDEA* (Hs00154455_m1), *CITED1* (Hs00918445_g1), *FABP4* (Hs01086177_m1), *PPARA* (Hs00947536_m1), *PPARG* (Hs0115513_m1), *PPARGC1A* (PGC-1α; Hs01084772_m1), *PRDM16* (Hs00223161_m1), *SLC2A4* (GLUT4; Hs00168966_m1), *TBX1* (Hs00962558_g1), and *UCP1* (Hs01084772_m1). The RT-PCR reaction was carried out using the QuantiTect Probe RT-PCR Kit (QIAGEN) in a thermal cycler (MiniAmp Plus, Thermo Fisher Scientific) under the following conditions: 50 °C for 30 min, 95 °C for 15 min, followed by 50 cycles of 94 °C for 15 s and 60 °C for 60 s. The mRNA levels of all genes were normalized to those of the internal control (*ACTB*).

### Immunofluorescence analysis

Preadipocyte cells were differentiated with or without falcarindiol (1, 3, 10, or 30 μM) in PhenoPlate-96 microplates (PerkinElmer, Waltham, USA) under the conditions described above. On day 8, cells were fixed with 4% paraformaldehyde in PBS for 20 min at room temperature, followed by three washes with DPBS. After washing, the cells were blocked and permeabilized with 3% BSA and 0.1% Triton-X 100 in DPBS for 30 min at room temperature. Subsequently, the cells were incubated with anti-UCP1 antibody (Cat No. ab10983, Cambridge, MA, USA) at 1/500 dilution in 3% BSA/DPBS overnight at 4 °C, followed by three washes with 3% BSA/DPBS. The cells were then incubated with goat anti-rabbit IgG (H + L) cross-adsorbed secondary antibody (Alexa Fluor 594) at 1/500 dilution, Hoechst 33,342 at 1/1000 dilution, and 1 μM BODIPY 493/503 in 3% BSA/DPBS for 2 h at room temperature. After washing three times with DPBS, fluorescent images were acquired using an Operetta CLS high-content analysis system (PerkinElmer). The fluorescence intensity and number of fluorescence-positive cells per well were quantified using Harmony software (version 4.9, PerkinElmer).

### Oxygen consumption rate measurement

Preadipocyte cells were differentiated with or without falcarindiol (1, 3, 10, or 30 μM) in Seahorse XFe96/XF Pro Cell Culture Microplates (Agilent) under the conditions described above. On day 8, the oxygen consumption rate (OCR) was measured using a Seahorse XF Cell Mito Stress Test Kit (Agilent) and Seahorse XFe96 extracellular flux analyzer (Agilent), according to the manufacturer’s instructions. The medium was replaced with Seahorse XF base medium supplemented with 10 mM glucose, 2 mM glutamine, and 1 mM pyruvate, and the cells were incubated at 37 °C in a non-CO_2_ incubator (HB-100N; TAITEC, Saitama, JAPAN) for 1 h. OCR measurements were performed following the sequential addition of oligomycin, carbonyl cyanide-4 (trifluoromethoxy) phenylhydrazone (FCCP), and rotenone/antimycin A to final concentrations of 2, 1, and 1 μM, respectively. The data were analyzed using Seahorse Wave Desktop software (version 2.6.3, Agilent).

### Statistical analysis

Data are presented as mean ± standard error of the mean (SEM). All statistical analyses were performed using EZR (version 1.68, Saitama Medical Center, Jichi Medical University, Saitama, Japan), a graphical user interface for R (version 4.3.1, The R Foundation for Statistical Computing, Vienna, Austria). Data were analyzed using one-way analysis of variance (ANOVA) followed by Dunnett’s post-hoc test. Statistical significance was set at* p* < 0.05.

## Results

### Falcarindiol affects the cell viability of human preadipocyte-derived adipocytes

We first investigated the effect of co-treatment with falcarindiol and human preadipocytes during adipogenic differentiation on cell viability. Preadipocytes were induced to differentiate for 8 days in the presence of 1–100 μM falcarindiol. Falcarindiol had no significant effect on cell proliferation at 1 and 3 μM but significantly increased cell viability by 9 and 14% at 10 and 30 μM, respectively. In contrast, 100 μM falcarindiol significantly reduced the cell viability by approximately 87% (Fig. 1b). Falcarindiol did not cause cytotoxicity in human preadipocytes at concentrations ≤ 30 μM. Therefore, in subsequent tests, falcarindiol was evaluated at concentrations ≤ 30 μM.

### Falcarindiol upregulates PPARγ target gene expression in human preadipocyte-derived adipocytes

**Fig. 2:**
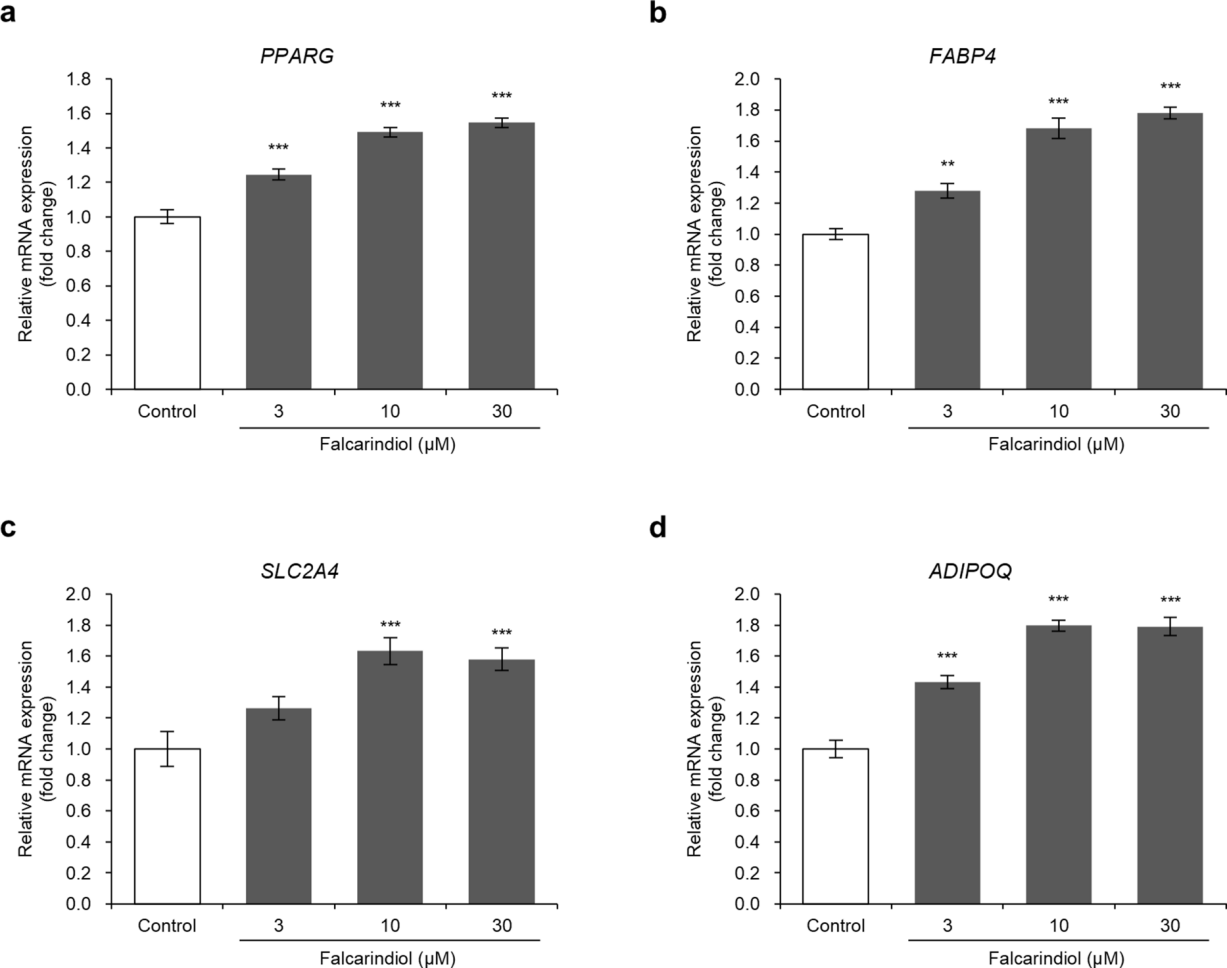
Effects of falcarindiol on the expression of PPARγ target genes in human preadipocyte-derived adipocytes. **a**–**d** Relative mRNA expression of *PPARG*, *FABP4*, *SLC2A4*, and *ADIPOQ*. Human preadipocytes were differentiated into mature adipocytes for eight days in differentiation medium with or without falcarindiol. Total RNA was isolated on day 8 and used for qRT-PCR analysis. The mRNA expression of each gene was normalized to that of *ACTB* and expressed as fold-induction relative to the control. Data are presented as mean ± SEM (n = 5). **p < 0.01 and ***p < 0.001 compared with the control (0.1% DMSO)

To confirm that falcarindiol acts as a PPARγ agonist, we evaluated the effect of falcarindiol on the expression of PPARγ target genes. Falcarindiol significantly increased *PPARG* and *FABP4* mRNA expression in a dose-dependent manner in the range of 3–30 μM (Fig. 2a, b). Although not significant at 3 μM, *SLC2A4* mRNA expression significantly increased at 10 and 30 μM falcarindiol (Fig. 2c). *ADIPOQ* mRNA expression significantly increased at 3–30 μM falcarindiol (Fig. 2d).

### Falcarindiol promotes beige adipocyte-related gene expression in human preadipocyte-derived adipocytes

**Fig. 3:**
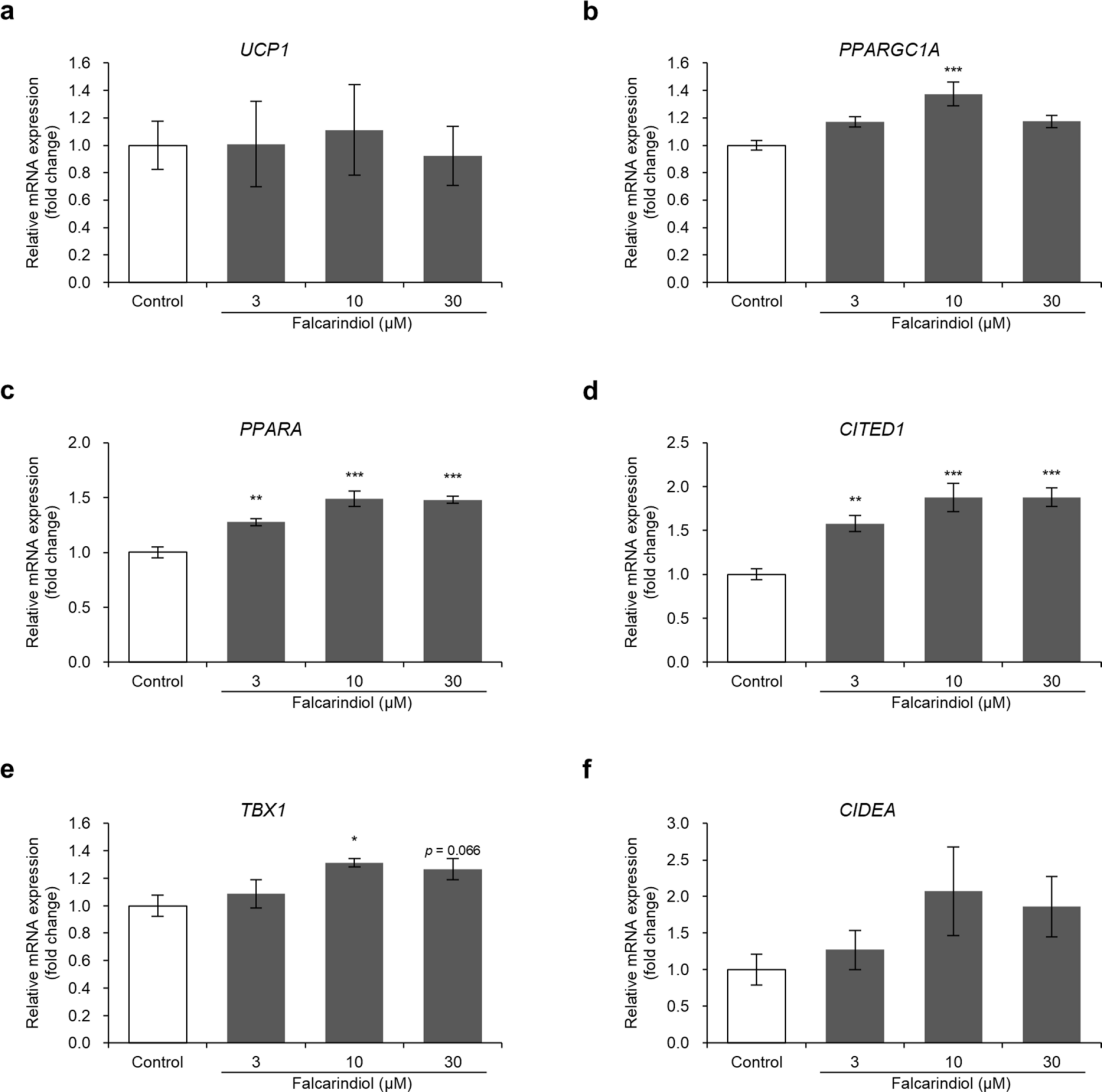
Effects of falcarindiol on the expression of beige adipocyte-related genes in human preadipocyte-derived adipocytes. **a**–**f** Relative mRNA expression of *UCP1*, *PPARGC1A*, *PPARA*, *CITED1*, *TBX1*, and *CIDEA*. Human preadipocytes were differentiated into mature adipocytes for 8 days in differentiation medium with or without falcarindiol. Total RNA was isolated on day 8 and used for qRT-PCR. The mRNA expression of each gene was normalized to that of *ACTB* and expressed as fold-induction relative to the control. Data are presented as mean ± SEM (n = 5). *p < 0.05, **p < 0.01, and ***p < 0.001 compared with the control (0.1% DMSO)

PPARγ agonists induce the browning of white adipocytes. Therefore, to confirm whether falcarindiol induces conversion of white adipocytes to the beige phenotype, we examined the expression of beige-adipocyte-related genes. Falcarindiol did not increase the mRNA expression of the *UCP-1* in the range of 3–30 μM (Fig. 3a). In contrast, falcarindiol increased the mRNA expression of *PPARGC1A* at 10 μM (Fig. 3b). In addition, it significantly increased the mRNA expression of *PPARA* and *CITED1* in a dose-dependent manner (Fig. 3c, d) and significantly increased the expression of *TBX1* at 10 μM (Fig. 3e). However, falcarindiol slightly, but not significantly, increased *CIDEA* mRNA expression (Fig. 2f).

### Falcarindiol promotes the differentiation and UCP1 expression in human preadipocyte-derived adipocytes

**Fig. 4:**
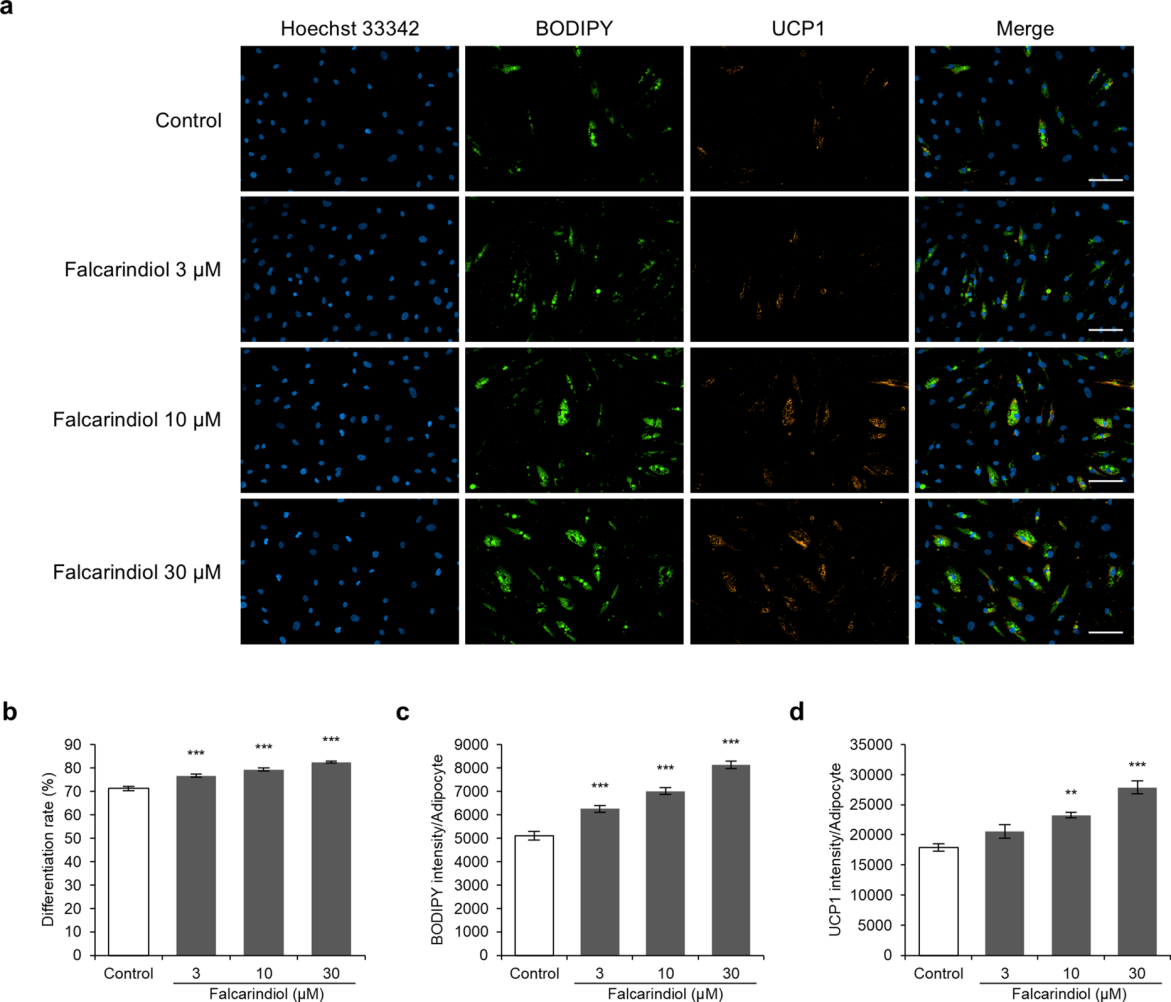
Effects of falcarindiol on differentiation and UCP-1 expression in human preadipocyte-derived adipocytes. Human preadipocytes were differentiated into mature adipocytes for 8 days in differentiation medium with or without falcarindiol. **a** Immunofluorescence staining of nuclei, lipid droplets, and UCP1. Cells were fixed on day 8 and stained with Hoechst 33,342 for nuclei (blue), BODIPY 493/503 for lipid droplets (green), or immunofluorescence for UCP1 (red). Fluorescent images were captured at 10 × magnification. Scale bars represent 100 μm. **b** Adipocyte differentiation rate was calculated as the ratio of BODIPY-positive cells to Hoechst-positive cells. **c**, **d** BODIPY and UCP1 intensities are expressed as values relative to the number of BODIPY-positive cells. **p < 0.01 and ***p < 0.001 compared with the control (0.1% DMSO). (Colour figure online)

Falcarindiol was confirmed to increase the expression of beige adipocyte-related genes, therefore, we evaluated the expression of the UCP1 protein using immunostaining to confirm differentiation into beige adipocytes (Fig. 4a). During the differentiation induction period, co-treatment with falcarindiol significantly increased the differentiation rate into mature adipocytes that accumulated lipid droplets in a dose-dependent manner (Fig. 4b). In addition, falcarindiol significantly increased fat accumulation in differentiated mature adipocytes in a dose-dependent manner (Fig. 4c). Furthermore, falcarindiol significantly increased the fluorescence intensity of UCP1 in mature adipocytes at 10 and 30 μM (Fig. 4d).

### Falcarindiol enhances the mitochondrial oxidative function in human preadipocyte-derived adipocytes

**Fig. 5:**
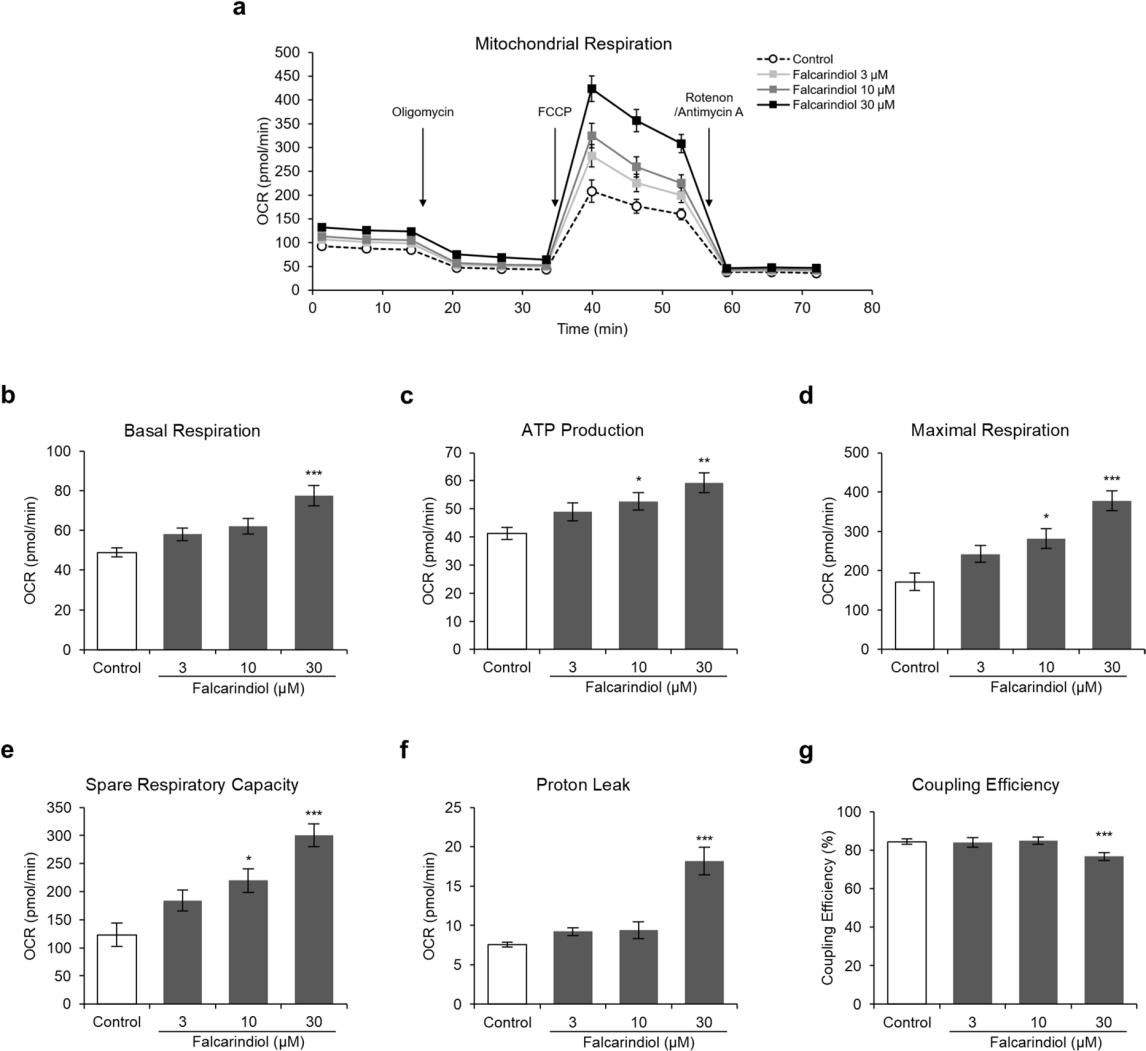
Effects of falcarindiol on mitochondrial respiration in human preadipocyte-derived adipocytes. Human preadipocytes were differentiated into mature adipocytes for eight days in differentiation medium with or without falcarindiol and the oxygen consumption rate (OCR) was measured using a Seahorse XFe96 extracellular flux analyzer. **a** Mitochondrial OCR curves with and without falcarindiol treatment. The mitochondrial respiration inhibitors oligomycin, FCCP, and rotenone/antimycin A were added during the measurement as indicated. **b** Basal respiration. **c** ATP production. **d** Maximal respiration. **e** Spare respiratory capacity. **f** Proton leak. **g** Coupling efficiency. Data are presented as mean ± SEM (n = 5). *p < 0.05, **p < 0.01, and ***p < 0.001 compared with thr control (0.1% DMSO)

To determine whether induction of UCP1 expression by falcarindiol leads to an increase in mitochondrial respiration, we measured the OCR in a mitochondrial stress test using a Seahorse analyzer. The cells treated with falcarindiol showed higher mitochondrial respiration than that of control cells (Fig. 5a). Specifically, falcarindiol increased basal respiration in a dose-dependent manner and was significantly increased at 30 μM compared with that in the control (Fig. 5b). In addition, falcarindiol increased ATP production in a dose-dependent manner, with significant increases at 10 and 30 μM. ATP production was measured following the addition of the ATP synthesis inhibitor oligomycin (Fig. 5c). Maximal respiration, measured following the addition of the uncoupler FCCP, also significantly increased (Fig. 5d), as did the spare respiratory capacity (Fig. 5e). Furthermore, 30 μM falcarindiol significantly increased proton leakage, induced by the mitochondrial complex rotenone/antimycin A (Fig. 5f), and significantly decreased coupling efficiency (Fig. 5g).

## Discussion

Adipocyte browning and mitochondrial biogenesis are important strategies for the prevention of obesity and subsequent chronic diseases. Previous studies have reported that various dietary compounds, such as naringenin (Rebello et al. 2019), 6-gingerol (Wang et al. 2019), and curcumin (Zhao et al. 2021), induce UCP1 expression in adipocytes, promote their browning, and improve mitochondrial respiration. Falcarindiol induces adipocyte differentiation and promotes glucose uptake in adipocytes (Atanasov et al. 2013; El-Houri et al. 2015). A more recent study showed that long-term administration of carrot powder containing falcarinol and falcarindiol to high-fat diet-induced obese mice improved oral glucose tolerance and increased the diversity and composition of the gut microbiota (Kobaek-Larsen et al. 2024). However, the effect of falcarindiol on fat browning had not been clarified. In this study, we revealed that one of the mechanisms by which falcarindiol prevents obesity is by increasing UCP1 expression and mitochondrial respiration in human preadipocyte-derived adipocytes.

Falcarindiol is an aliphatic C_17_-polyacetylene and inhibits the cell proliferation of human cancer cells (Purup et al. 2009; Jin et al. 2012; Wang et al. 2013). Cicutoxin toxicity (the aliphatic C_17_-polyacetylene and violent toxins) is due to the π-bond conjugation system and geometry of the double bonds; however, the terminal O-functional group and allylic alcohol are essential (Konoshima and Lee 1986; Uwai et al. 2000; Lin et al. 2016). Falcarinol and falcarindiol do not have these structures. Falcarinol has an antiproliferative effect, and the diyne functionality is essential for this activity. Additionally, the configuration of asymmetric centers and the length of the aliphatic carbon chain may be important (Christensen 2020). The antiproliferative effect of falcarindiol is weaker than that of falcarinol (Purup et al. 2009; Zaini et al. 2012). Furthermore, falcarinol-type polyacetylenes have a biphasic effect (hormesis) on cell proliferation, with an increase at relatively low concentrations and a decrease at high concentrations (Hansen et al. 2003; Young et al. 2007; Purup et al. 2009). Consistent with these reports, we confirmed that falcarindiol was cytotoxic to human preadipocytes at 100 µM or higher but was non-cytotoxic and promoted cell proliferation at 30 µM or lower.

Previous research has shown that falcarindiol activates PPARγ. However, falcarindiol blocks PPARγ activation when co-treated with pioglitazone, a thiazolidine PPARγ agonist. Therefore, it acts as a partial agonist for PPARγ (Atanasov et al. 2013). Falcarindiol significantly increases the expression of *aP2* (*FABP4*), a PPARγ target gene, in 3T3-L1 adipocytes, resulting in the promotion of adipocyte differentiation (El-Houri et al. 2015). In the present study, we demonstrated that treatment with falcarindiol during differentiation increased the gene expression of PPARγ target genes, such as *PPARG*, *FABP4*, *SLC2A4,* and *ADIPOQ*, in adipocytes derived from human primary cultured cells. Furthermore, falcarindiol increased the fat accumulation and promoted differentiation in adipocytes derived from human primary cultured cells. Similarly, the PPARγ agonist, rosiglitazone, increases the mRNA expression of *PPARG*, *ADIPOQ*, and *FABP4* in human adipose-derived stromal/progenitor cells (Bartesaghi et al. 2015). These suggest that falcarindiol acts as a PPARγ agonist in human primary cultured adipocytes.

Rosiglitazone induces the browning of adipocytes through stabilization of the PRDM16 protein (Ohno et al. 2012) and increases the mRNA expression of *UCP1*, *PGC1α*, *CITED1*, and *TBX1* in human adipose-derived stromal/progenitor cells (Bartesaghi et al. 2015). Tesaglitazar, a PPARα and PPARγ agonist, also increases the mRNA expression of *UCP1*, *PGC1α*, and *PPARα* in mouse and human white adipocytes (Kroon et al. 2020). Consistently, in the current study, falcarindiol increased the expression of beige adipocyte-related genes, such as *PPARGC1A*, *PPARA*, *CITED1*, *TBX1*, and *CIDEA*. However, the UCP1 protein level was significantly upregulated, but its mRNA expression did not significantly increase. *UCP1* mRNA expression increases rapidly in response to cold stimulation and then decreases, but protein expression continues to increase. This may be related to the half-life of the UCP1 protein (Nedergaard and Cannon 2013). The fact that mRNA and protein expression were assessed during the same differentiation period may explain why no significant increase in *UCP1* mRNA expression was observed in this study. The increase in UCP1 protein levels in this study may indicate that falcarindiol promoted UCP1 expression and beige adipogenesis.

Beige adipocytes have higher basal respiration, maximal respiration, and uncoupled respiration than white adipocytes (Loft et al. 2015). Increased UCP1 protein levels promote uncoupled respiration through proton leakage (Kajimura et al. 2015). Therefore, to confirm whether the beige adipocyte phenotype was induced, we evaluated the effect of falcarindiol on mitochondrial respiration by analyzing OCR. As a result, falcarindiol increased basal respiration, ATP-linked respiration, maximum respiration, and spare capacity in mitochondria. In addition, falcarindiol increased proton-leak OCR and decreased coupling efficiency. Co-stimulation of rosiglitazone with β-adrenergic agonists promotes the UCP1 protein levels and mitochondrial respiration, including basal and dissociative respiration, and the increase in rosiglitazone-induced dissociative respiration is significantly reduced by UCP1 knockdown (Khor et al. 2019). This indicates that agonist-induced PPARγ activation promotes uncoupling respiration via induction of UCP1 expression. Therefore, the promotion of uncoupling respiration by falcarindiol may be due to the induction of UCP1.

The present study demonstrates that falcarindiol promotes beige adipocyte-related gene expression and mitochondrial respiration; however, there are several limitations to this study. The induction of the beige adipocyte-like phenotype confirmed in this study is likely to be due to the activation of PPARγ, since falcarindiol has been reported to act as a PPARγ agonist (Atanasov et al. 2013; El-Houri et al. 2015); however, it is still possible that this is due to a pathway other than PPARγ activation. Capsinoids (Ohyama et al. 2016), fish oil (Kim et al. 2015), and the linoleic acid metabolite (Kim et al. 2017) induce adipocyte browning via the sympathetic nervous system (SNS). These food-derived components are thought to activate the SNS by binding to transient receptor potential vanilloid 1 (TRPV1), with the subsequent release of noradrenaline and its binding to β3-adrenegic receptors (β3-AR) on adipocytes, triggering adipocyte browning. The binding of falcarindiol to TRPV1 has not been investigated; therefore, it is possible that falcarindiol induces adipocyte browning via this pathway. To clarify the detailed mechanism of falcarindiol on adipocyte browning, it is necessary to evaluate falcarindiol in combination with PPARγ antagonists and β3-AR inhibitors, as well as elucidating the signaling pathways involved. In addition, falcarindiol has been shown to enhance mitochondrial respiration, but it remains unclear whether this is due to an increase in mitochondrial biogenesis and/or changes in mitochondrial function. Furthermore, cytotoxicity was observed at a concentration of 100 μM falcarindiol in this study; thus, cell evaluation was conducted at concentrations of 30 μM or less. However, further verification is necessary to determine the optimal concentration within the 30–100 μM range with the highest efficacy.

This in vitro study revealed that falcarindiol promotes beige adipocyte-related gene expression and mitochondrial respiration, but in vivo and clinical verification are required. To the best of our knowledge, there are no reports on the concentration of falcarindiol in the body in animal or human studies; however, the maximum concentration of falcarinol, an 8-dehydroxy compound of falcarindiol, in plasma after the ingestion of 900 mL of carrot juice (containing 49 μmol falcarinol) is 0.01 μM (Brandt et al. 2004; Christensen and Brandt 2006). Compared with previous reports, the effective concentration of falcarindiol in the current in vitro study is 10–30 μM, which is a large difference in concentration. As falcarindiol is a lipophilic compound, it is expected to accumulate in relatively large amounts in adipose tissue. The concentration of falcarindiol evaluated in this study is believed to be related to its biological effects, but further in vivo and clinical studies are needed to determine whether falcarindiol exerts an anti-obesity effect by activating PPARγ and inducing adipocyte differentiation in the body.

## Conclusion

Our results revealed that falcarindiol promotes a beige adipocyte-like phenotype and mitochondrial respiration in human preadipocyte-derived adipocytes. The present findings suggest that the consumption of Apiaceae plants may have an anti-obesity effect. Further verification through in vivo and clinical studies is needed to clarify the effects of falcarindiol and Apiaceae vegetables.

## Data Availability

No datasets were generated or analysed during the current study.

## References

[CR1] Alfurayhi R, Huang L, Brandt K (2023) Pathways affected by falcarinol-type polyacetylenes and implications for their anti-inflammatory function and potential in cancer chemoprevention. Foods. 10.3390/foods12061192 10.3390/foods12061192 PMC10048309 36981118

[CR2] Atanasov AG, Blunder M, Fakhrudin N et al (2013) Polyacetylenes from *Notopterygium incisum* - new selective partial agonists of peroxisome proliferator-activated receptor-gamma. PLoS ONE. 10.1371/journal.pone.0061755 23630612 10.1371/journal.pone.0061755 PMC3632601

[CR3] Bartesaghi S, Hallen S, Huang L et al (2015) Thermogenic activity of UCP1 in human white fat-derived beige adipocytes. Mol Endocrinol 29:130–139. 10.1210/me.2014-1295 25389910 10.1210/me.2014-1295 PMC5414770

[CR4] Brandt K, Christensen LP, Hansen-Møller J et al (2004) Health promoting compounds in vegetables and fruits: a systematic approach for identifying plant components with impact on human health. Trends Food Sci Technol 15:384–393. 10.1016/j.tifs.2003.12.003

[CR5] Chouchani ET, Kazak L, Spiegelman BM (2019) New advances in adaptive thermogenesis: UCP1 and beyond. Cell Metab 29:27–37. 10.1016/j.cmet.2018.11.002 30503034 10.1016/j.cmet.2018.11.002

[CR6] Christensen LP (2011) Aliphatic C 17-polyacetylenes of the falcarinol type as potential health promoting compounds in food plants of the Apiaceae family. Recent Pat Food Nutr Agric 3:64–77. 10.2174/2212798411103010064 21114468 10.2174/2212798411103010064

[CR7] Christensen LP (2020) Bioactive C17 and C18 acetylenic oxylipins from terrestrial plants as potential lead compounds for anticancer drug development. Molecules. 10.3390/molecules25112568 32486470 10.3390/molecules25112568 PMC7321150

[CR8] Christensen LP, Brandt K (2006) Bioactive polyacetylenes in food plants of the Apiaceae family: occurrence, bioactivity and analysis. J Pharm Biomed Anal 41:683–693. 10.1016/j.jpba.2006.01.057 16520011 10.1016/j.jpba.2006.01.057

[CR9] Dawid C, Dunemann F, Schwab W, Nothnagel T, Hofmann T (2015) Bioactive C17-polyacetylenes in carrots (*Daucus carota* L.): current knowledge and future perspectives. J Agric Food Chem 63:9211–9222. 10.1021/acs.jafc.5b04357 26451696 10.1021/acs.jafc.5b04357

[CR10] Deding U, Baatrup G, Christensen LP, Kobaek-Larsen M (2020) Carrot intake and risk of colorectal cancer: a prospective cohort study of 57,053 Danes. Nutrients. 10.3390/nu12020332 32012660 10.3390/nu12020332 PMC7071341

[CR11] Deding U, Baatrup G, Kaalby L, Kobaek-Larsen M (2023) Carrot intake and risk of developing cancer: a prospective cohort study. Nutrients 15:678. 10.3390/nu15030678 36771385 10.3390/nu15030678 PMC9919376

[CR12] El-Houri RB, Kotowska D, Christensen KB et al (2015) Polyacetylenes from carrots (*Daucus carota*) improve glucose uptake in vitro in adipocytes and myotubes. Food Funct 6:2135–2144. 10.1039/c5fo00223k 25970571 10.1039/c5fo00223k

[CR13] Hansen SL, Purup S, Christensen LP (2003) Bioactivity of falcarinol and the influence of processing and storage on its content in carrots (*Daucus carota* L). J Sci Food Agric 83:1010–1017. 10.1002/jsfa.1442

[CR14] Ikeda K, Maretich P, Kajimura S (2018) The common and distinct features of brown and beige adipocytes. Trends Endocrinol Metab 29:191–200. 10.1016/j.tem.2018.01.001 29366777 10.1016/j.tem.2018.01.001 PMC5826798

[CR15] Jin HR, Zhao J, Zhang Z et al (2012) The antitumor natural compound falcarindiol promotes cancer cell death by inducing endoplasmic reticulum stress. Cell Death Dis. 10.1038/cddis.2012.122 22914324 10.1038/cddis.2012.122 PMC3434669

[CR16] Kajimura S, Spiegelman BM, Seale P (2015) Brown and beige fat: physiological roles beyond heat generation. Cell Metab 22:546–559. 10.1016/j.cmet.2015.09.007 26445512 10.1016/j.cmet.2015.09.007 PMC4613812

[CR17] Khor NWM, Swarbrick MM, Gunton JE (2019) Inducible UCP1 silencing: a lentiviral RNA-interference approach to quantify the contribution of beige fat to energy homeostasis. PLoS ONE. 10.1371/journal.pone.0223987 31751350 10.1371/journal.pone.0223987 PMC6872148

[CR18] Kim M, Goto T, Yu R et al (2015) Fish oil intake induces UCP1 upregulation in brown and white adipose tissue via the sympathetic nervous system. Sci Rep 5:18013. 10.1038/srep18013 26673120 10.1038/srep18013 PMC4682086

[CR19] Kim M, Furuzono T, Yamakuni K et al (2017) 10-oxo-12(Z)-octadecenoic acid, a linoleic acid metabolite produced by gut lactic acid bacteria, enhances energy metabolism by activation of TRPV1. FASEB J 31:5036–5048. 10.1096/fj.201700151R 28754711 10.1096/fj.201700151R

[CR20] Klein S, Gastaldelli A, Yki-Järvinen H, Scherer PE (2022) Why does obesity cause diabetes? Cell Metab 34:11–20. 10.1016/j.cmet.2021.12.012 34986330 10.1016/j.cmet.2021.12.012 PMC8740746

[CR21] Kobaek-Larsen M, El-Houri RB, Christensen LP, Al-Najami I, Fretté X, Baatrup G (2017) Dietary polyacetylenes, falcarinol and falcarindiol, isolated from carrots prevents the formation of neoplastic lesions in the colon of azoxymethane-induced rats. Food Funct 8:964–974. 10.1039/c7fo00110j 28197615 10.1039/c7fo00110j

[CR22] Kobaek-Larsen M, Baatrup G, Notabi MK et al (2019) Dietary polyacetylenic oxylipins falcarinol and falcarindiol prevent inflammation and colorectal neoplastic transformation: a mechanistic and dose-response study in a rat model. Nutrients. 10.3390/nu11092223 31540047 10.3390/nu11092223 PMC6769548

[CR23] Kobaek-Larsen M, Maschek S, Kolstrup SH et al (2024) Effect of carrot intake on glucose tolerance, microbiota, and gene expression in a type 2 diabetes mouse model. Clin Transl Sci. 10.1111/cts.70090 39625861 10.1111/cts.70090 PMC11613996

[CR24] Konoshima T, Lee K-H (1986) Cicutoxin, an antileukemic principle from *Cicuta maculata*, and the cytotoxicity of the related derivatives. J Nat Prod 49:1117–1121 3572419 10.1021/np50048a028

[CR25] Kroon T, Harms M, Maurer S et al (2020) PPARγ and PPARα synergize to induce robust browning of white fat in vivo. Mol Metab. 10.1016/j.molmet.2020.02.007 32248079 10.1016/j.molmet.2020.02.007 PMC7132097

[CR26] Lin M, Zhang W, Su J (2016) Toxic polyacetylenes in the genus *Bupleurum* (Apiaceae)—distribution, toxicity, molecular mechanism and analysis. J Ethnopharmacol 193:566–573. 10.1016/j.jep.2016.09.052 27693772 10.1016/j.jep.2016.09.052

[CR27] Loft A, Forss I, Siersbæk MS et al (2015) Browning of human adipocytes requires KLF11 and reprogramming of PPARγ superenhancers. Genes Dev 29:7–22. 10.1101/gad.250829.114 25504365 10.1101/gad.250829.114 PMC4281566

[CR28] Metzger BT, Barnes DM, Reed JD (2008) Purple carrot (*Daucus carota* L.) polyacetylenes decrease lipopolysaccharide-induced expression of inflammatory proteins in macrophage and endothelial cells. J Agric Food Chem 56:3554–3560. 10.1021/jf073494t 18433135 10.1021/jf073494t

[CR29] Nedergaard J, Cannon B (2013) UCP1 mRNA does not produce heat. Biochim Biophys Acta Mol Cell Biol Lipids 1831:943–949. 10.1016/j.bbalip.2013.01.009 10.1016/j.bbalip.2013.01.009 23353596

[CR30] Ohno H, Shinoda K, Spiegelman BM, Kajimura S (2012) PPARγ agonists induce a white-to-brown fat conversion through stabilization of PRDM16 protein. Cell Metab 15:395–404. 10.1016/j.cmet.2012.01.019 22405074 10.1016/j.cmet.2012.01.019 PMC3410936

[CR31] Ohyama K, Nogusa Y, Shinoda K, Suzuki K, Bannai M, Kajimura S (2016) A synergistic antiobesity effect by a combination of capsinoids and cold temperature through promoting beige adipocyte biogenesis. Diabetes 65:1410–1423. 10.2337/db15-0662 26936964 10.2337/db15-0662 PMC4839206

[CR32] Powell-Wiley TM, Poirier P, Burke LE et al (2021) Obesity and cardiovascular disease a scientific statement from the American heart association. Circulation 143:E984–E1010. 10.1161/cir.0000000000000973 33882682 10.1161/CIR.0000000000000973 PMC8493650

[CR33] Purup S, Larsen E, Christensen LP (2009) Differential effects of falcarinol and related aliphatic c-17-polyacetylenes on intestinal cell proliferation. J Agric Food Chem 57:8290–8296. 10.1021/jf901503a 19694436 10.1021/jf901503a PMC2745230

[CR34] Rebello CJ, Greenway FL, Lau FH et al (2019) Naringenin promotes thermogenic gene expression in human white adipose tissue. Obesity (Silver Spring) 27:103–111. 10.1002/oby.22352 30506905 10.1002/oby.22352 PMC6309263

[CR35] Romieu I, Dossus L, Barquera S et al (2017) Energy balance and obesity: what are the main drivers? Cancer Causes Control 28:247–258. 10.1007/s10552-017-0869-z 28210884 10.1007/s10552-017-0869-z PMC5325830

[CR36] Uwai K, Ohashi K, Takaya Y et al (2000) Exploring the structural basis of neurotoxicity in C17-polyacetylenes isolated from water hemlock. J Med Chem 43:4508–4515. 10.1021/jm000185k 11087575 10.1021/jm000185k

[CR37] Venkatesan T, Choi YW, Lee J, Kim YK (2018) Falcarindiol inhibits LPS-induced inflammation via attenuating MAPK and JAK-STAT signaling pathways in murine macrophage RAW 264.7 cells. Mol Cell Biochem 445:169–178. 10.1007/s11010-017-3262-z 29368095 10.1007/s11010-017-3262-z

[CR38] Wang CZ, Zhang Z, Huang WH et al (2013) Identification of potential anticancer compounds from *Oplopanax horridus*. Phytomedicine 20:999–1006. 10.1016/j.phymed.2013.04.013 23746754 10.1016/j.phymed.2013.04.013 PMC3729876

[CR45] Wang J, Zhang L, Dong L, et al (2019) 6-Gingerol, a Functional Polyphenol of Ginger, Promotes Browning through an AMPK-Dependent Pathway in 3T3-L1 Adipocytes. J Agric Food Chem 67:14056–14065. 10.1021/acs.jafc.9b05072 10.1021/acs.jafc.9b05072 31789021

[CR39] Wang XJ, Luo Q, Li T et al (2022) Origin, evolution, breeding, and omics of Apiaceae: a family of vegetables and medicinal plants. Hortic Res. 10.1093/hr/uhac076 38239769 10.1093/hr/uhac076 PMC10795576

[CR40] World Obesity Federation (2023) World obesity atlas 2023. https://www.worldobesity.org/resources/resource-library/world-obesity-atlas-2023. Accessed 9 Apr 2025

[CR41] Yoshida J, Seino H, Ito Y et al (2013) Inhibition of glycogen synthase kinase-3β by falcarindiol isolated from Japanese parsley (*Oenanthe javanica*). J Agric Food Chem 61:7515–7521. 10.1021/jf401042m 23895038 10.1021/jf401042m

[CR42] Young JF, Duthie SJ, Milne L, Christensen LP, Duthie GG, Bestwick CS (2007) Biphasic effect of falcarinol on CaCo-2 cell proliferation, DNA damage, and apoptosis. J Agric Food Chem 55:618–623. 10.1021/jf0616154 17263451 10.1021/jf0616154

[CR43] Zaini RG, Brandt K, Clench MR, Le Maitre CL (2012) Effects of bioactive compounds from carrots (*Daucus carota* L.), polyacetylenes, beta-carotene and lutein on human lymphoid leukaemia cells. Anticancer Agents Med Chem 12:640–652. 10.2174/187152012800617704 22263789 10.2174/187152012800617704

[CR44] Zhao D, Pan Y, Yu N et al (2021) Curcumin improves adipocytes browning and mitochondrial function in 3T3-L1 cells and obese rodent model. R Soc Open Sci. 10.1098/rsos.200974 33959308 10.1098/rsos.200974 PMC8074937

